# The association between cross-cultural competence and well-being among registered native and foreign-born nurses in Finland

**DOI:** 10.1371/journal.pone.0208761

**Published:** 2018-12-07

**Authors:** Karolina Wesołowska, Laura Hietapakka, Marko Elovainio, Anna-Mari Aalto, Anu-Marja Kaihlanen, Tarja Heponiemi

**Affiliations:** 1 Department of Social and Health Systems Research, National Institute for Health and Welfare, Helsinki, Finland; 2 Department of Psychology and Logopedics, Faculty of Medicine, University of Helsinki, Helsinki, Finland; Tampereen Yliopisto, FINLAND

## Abstract

**Background:**

A growing body of research indicates that cross-cultural competence in nurses can improve migrant patients’ health-related outcomes, but little is known about the potential benefits of cross-cultural competence on the nurses’ own well-being.

**Objective:**

To examine whether cross-cultural competence (empathy, skills, positive attitudes, and motivation) is associated with perceived time pressure at work, psychological distress, and sleep problems among registered nurses in Finland, and whether there are differences in these potential associations between native and foreign-born nurses.

**Methods:**

The present cross-sectional study was based on a sample of 212 foreign-born nurses licensed to practice in Finland and a random sample of 744 native Finnish nurses. Data were collected with a questionnaire and analyzed using multiple linear regression and structural equation modeling (SEM).

**Results:**

Of all four dimensions of cross-cultural competence, only empathy was associated with perceived time pressure (β = –0.13, *p* = .018), distress (β = –0.23, *p* < .001), and sleep problems (β = –0.14, *p* = .004) after the adjustment for gender, age, employment sector, and frequency of interacting with patients and colleagues from different cultures. There were no differences between native and foreign-born nurses in these observed associations (all *p*s > .05).

**Conclusions:**

Cross-cultural empathy may protect against perceived time pressure, distress, and sleep problems in both native and foreign-born nurses. Thus, the promotion of this component of cross-cultural competence among nursing personnel should be encouraged.

## Introduction

Due to an increase in the number of international migrants across the world over the last two decades (the number reached 258 million in 2017 [1]), populations of many European countries [2], including Finland [3], are becoming more culturally and ethnically diverse. This phenomenon also increases cultural diversity of patients and health care personnel. Between 2010 and 2011, foreign-born physicians and nurses accounted for more than 30% and 40%, respectively, of the health care workforce in some countries in Europe [4].

Working in a culturally diverse team and with patients of different cultural backgrounds can be challenging due to differences in opinions, beliefs, thoughts, norms, customs, and traditions. If poorly managed, cultural diversity can result in miscommunication [5], maladaptive behaviors [6], and interpersonal conflicts [7]. Thus, it has been widely suggested that native and foreign-born health care professionals need to be equipped with cross-cultural competence [8]. Cross-cultural competence has been defined as a set of skills (e.g., patience and cultural adaptability), attitudes (e.g., tolerance and respect towards other cultures), motivations (e.g., cultural curiosity), emotions (e.g., multicultural empathy and sensitivity), and knowledge (e.g., awareness of one’s own values and perceptions) that enable health care providers to effectively manage cultural differences in the workplace [9]. The concept has attracted increasing international attention in recent years mostly as a result of the recognition that discrimination, biases, and prejudices among health care providers [5, 10] as well as linguistic [5, 11] and cultural [5] barriers between health care professionals and patients can interfere the quality of care delivery.

An increasing body of literature (see a review in [12–13]) reports that cross-cultural competence in health care employees, including nurses, can improve migrant patients’ health-related outcomes, satisfaction, and trust, but little is known about the potential benefits of cross-cultural competence on the nurses’ own well-being. Cross-cultural competence has been shown to increase job satisfaction [14] and decrease the risk of burnout development [15] among nursing personnel. It is, however, unclear whether cross-cultural competence may protect against work-related risks of lower well-being, including perceived time pressure, or such early indicators of ill-being as psychological distress and sleep problems. These three are common problems in nurses [16–18], contributing to clinical burnout [19], major depressive disorder [20], turnover intention [21], and decreased quality of health care services [22], among others.

Previous studies have shown that multicultural empathy and flexibility, both closely linked to tolerant attitude towards other ethnic groups [23], are associated with problem-solving styles [24], known to reduce stress levels and to improve psychological adjustment [25]. Cross-cultural empathy has also been documented to relate to emotional intelligence [26], which promotes collaborative conflict management behaviors [27]. Further, there is evidence suggesting that positive emotional states [28] and emotional regulation [28–29] can have a positive effect on sleep quality. Thus, it seems possible that cross-cultural competence may decrease the risk of distress and sleep problems by facilitating better coping with challenging and sensitive cross-cultural situations, directly related to health outcomes. More effective coping with difficulties encountered in interaction with patients and colleagues from different cultures may also leave more time for the completion of actual work tasks. This, in turn, may result in a lower risk of perceived time pressure, which in itself has negatively been linked to well-being [30]. It has previously been shown that active, problem-focused coping interacts with perceived time pressure and work overload in the prediction of mental health [31]. Therefore, it is reasonable to assume that being culturally competent may also confer protection against perceived time pressure and, consequently, against distress and sleep problems, too.

Cross-cultural competence may be of particular importance for foreign-born nurses, given that most of their work colleagues and patients are from different cultural backgrounds and work culture can substantially differ from that which they have been used to. Good cross-cultural competence among migrant nurses, especially empathy, skills, and positive attitudes, may leave less space for the experience of discrimination, isolation, and difficulties in interactions with patients and colleagues by promoting efficient cross-cultural communication. For instance, there is some evidence suggesting that higher cross-cultural empathy [32–33], sensitivity [33], and skills [34] can be associated with more effective intercultural communication. Further, it has been found that cross-cultural empathy may help foreign-born physicians when encountering problems with patients from different cultures, decrease the likelihood of discrimination, and facilitate their integration into a foreign country [35]. Therefore, it is possible that cross-cultural competence may be especially beneficial to the well-being of foreign-born nurses.

The objective of the present cross-sectional study was to examine whether cross-cultural competence (empathy, skills, positive attitudes, and motivation) is associated with perceived time pressure at work, distress, and sleep problems among registered nurses in Finland, and whether there are differences in these potential associations between native and foreign-born nurses. The relationships were tested controlling, in addition to gender and age, for the possible confounding effects of employment sector and frequency of interacting with patients and colleagues from different cultures. These two factors have previously been shown to be associated with cross-cultural competence [36–37] and well-being indicators [38–40]. We hypothesized that:

H_1_–cross-cultural competence (empathy, skills, positive attitudes, and motivation) would be associated with perceived time pressure, distress, and sleep problems among registered nurses in Finland after adjusting for the potential confounders;

H_2_–there would be differences in these plausible associations between foreign-born and Finnish nurses, that is, these associations would be stronger for foreign-born nurses than for Finnish nurses.

## Materials and methods

### Participants

The data (S1 Dataset) collection was conducted during the fall of 2017 in Finland (14^th^ of September–5^th^ of November). Information on Finnish health care professionals stored in the Central Register (known as JulkiTerhikki) was delivered by Valvira (i.e., National Supervisory Authority for Welfare and Health). Two independent samples—of Finnish nurses and foreign-born nurses—were selected.

The Finnish nurses’ group included a random sample (*n* = 2001) drawn from the entire population of registered nurses in Finland (*N* = 114 668; data obtained from the Central Register). The selection criteria were as follows: born in or after 1950, licensed to practice in Finland, and having a postal address in Finland.

The Central Register included information on 617 foreign-trained nurses who met the above-mentioned criteria. Since this group could include native Finns who had studied abroad, those nurses whose mother tongue was one of the two official languages in Finland (Finnish or Swedish) were excluded (*n* = 128). Thereby, a sample of 489 foreign-born nurses was obtained.

Of these two samples, we managed to obtain e-mail or postal addresses for 1790 Finnish and 474 foreign-born nurses. An e-mail invitation including a link to the electronic questionnaire (available for the foreign-born nurses in Finnish (S1 Appendix), Swedish, Estonian, Russian, and English (S2 Appendix), and for the native nurses in Finnish and Swedish) was sent to those nurses whose e-mail addresses were obtained. To those participants for whom e-mail addresses were unavailable, a postal invitation to the electronic survey was sent. Two reminders were sent to those nurses who had not answered (the second also included a printed questionnaire available only in Finnish). There were 781 (43.6%) native nurses and 222 (46.8%) foreign-born nurses who filled in the questionnaire. Of the 781 native nurses, those who reported that their country of birth was not Finland were excluded (*n* = 26). Of the 222 foreign-born nurses, we excluded those who had been born in Finland (*n* = 3). Moreover, since the minimum retirement age in Finland is 63, nurses aged 63 and over were excluded (*n* = 18). Thus, the final study sample comprised 744 native nurses (91.0% women; *M*_age_ = 34.52, *SD* = 8.89, range 23–61) and 212 foreign-born nurses (94.3% women; *M*_age_ = 42.03, *SD* = 9.50, range 24–61).

Of the 956 nurses (both native and foreign-born), 155 (16.2%) had missing values in at least one of the variables used in the present study, 62 (6.5%)—in at least one of the dimensions of cross-cultural competence, 37 (3.9%)—in at least one of the outcome variables, and 79 (8.3%)—in at least one of the control variables. Individuals with complete data reported higher levels of perceived time pressure compared with those lost due to attrition (3.68 vs. 3.43; *t*(936) = 2.42, *p* = .016). The groups did not, however, differ in respect of gender (χ2(1) = 0.77, *p* = .382), age (36.04 vs. 36.96; *t*(954) = –1.10, *p* = .270), employment sector (χ2(2) = 5.48, *p* = .064), frequency of interacting with patients (*U* = 56056.5, *p* = .881) and colleagues (*U* = 39510.5, *p* = .948) from different cultures, levels of overall cross-cultural competence (3.69 vs. 3.70; *t*(892) = –0.20, *p* = .840), empathy (3.85 vs. 3.84; *t*(929) = 0.13, *p* = .900), skills (4.06 vs. 4.06; *t*(924) = –0.12, *p* = .908), positive attitudes (2.50 vs. 2.55; *t*(940) = –0.70, *p* = .481), motivation (3.96 vs. 3.97; *t*(931) = –0.16, *p* = .870), distress (1.93 vs. 1.98; *t*(943) = –0.71, *p* = .479), or sleep problems (2.48 vs. 2.45; *t*(938) = 0.33, *p* = .744). Missing values in all study variables were imputed using multiple imputation with chained equations (20 imputations [41]) for the purpose of the all multiple linear regression analyses (*n* = 956). In the structural equation modeling analysis (SEM; *n* = 881), full information maximum likelihood estimation was used to reduce the possibility of biased results due to missing data [42].

The study was approved by the ethics committee of the National Institute for Health and Welfare in Finland. Responding to the questionnaire was seen as a consent to participate. The survey script reminded the participants that they were under no obligation to complete and/or submit the survey.

### Measures

#### Cross-cultural competence

The Cross-Cultural Competence instrument for the Health Care Profession (CCCHP [9]) was used to measure cross-cultural competence. The CCCHP is a multidimensional tool composed of five dimensions of cross-cultural competence among health care professionals. These dimensions are: Cross-Cultural Motivation/Curiosity, Cross-Cultural Attitudes, Cross-Cultural Skills, Cross-Cultural Emotions/Empathy, and Cross-Cultural Knowledge/Awareness. The questionnaire consists of 27 items rated on a 5-point scale (1 = *fully disagree* and 5 = *fully agree*). In the Finnish version of the CCCHP (α = 0.88 [43]), which was used here, one item of the Cross-Cultural Motivation/Curiosity subscale *It is important to me to treat patients according to their cultural needs and individual values* was excluded from the instrument due to poor factor loading. The Cross-Cultural Knowledge/Awareness subscale was also excluded as it showed low reliability (α = 0.39 in nurses and α = 0.54 in physicians), similarly as in Bernhard’s original study [9]. Cross-Cultural Motivation/Curiosity includes eight items (e.g., *I find exciting to treat patients with a migration background*; α = 0.86), Cross-Cultural Attitudes—four items (e.g., *People who migrated to Finland should adapt to society*, *not the other way around*; α = 0.74), Cross-Cultural Skills—five items (e.g., *With patients who do not understand Finnish very well*, *I take more time to discuss their expectations and fears*; α = 0.80), and Cross-Cultural Emotions/Empathy—five items (e.g., *In my professional interaction with patients with a migration background*, *I often feel unsure and frustrated*; α = 0.79). Items of the Cross-Cultural Attitudes and the Cross-Cultural Emotion/Empathy subscales are presented in reverse order to prevent tendencies towards the response set. For the purpose of statistical analysis, these items were recoded so that higher values indicate more positive attitudes and emotional reactions towards other cultures and cultural diversity. Thereafter, the mean scores for the total scale (i.e., overall cross-cultural competence) and its subscales were computed.

The basic organization of the Finnish version of the CCCHP (i.e., the pattern of loadings of items on the latent factors and the degree to which each item contributes to the latent constructs) has been shown to be the same in native Finnish nurses and foreign-born physicians licensed to work in Finland [43], suggesting that the measure can be an appropriate tool for studies focused on the health care personnel in Finland. However, to ensure that the basic structure of cross-cultural competence was equivalent in native Finnish and foreign-born nurses included in our study sample, we performed measurement invariance tests using semTools in R statistical software. The results of this additional analysis confirmed the configural and metric invariance of cross-cultural competence across the two groups (S1 Table).

#### Perceived time pressure

Time pressure was measured by two items (α = 0.90) derived from the Nurse Stress Index (NSI [44–45]). These items were: *Constant rush and pressure due to uncompleted work* and *Not enough time to perform work properly*. The respondents were asked to rate to what extent these issues had disturbed, worried, or stressed them in their job during the last six months on a 5-point scale (ranging from 1 = *hardly ever* to 5 = *very often or continuously*). We then calculated the mean scores.

#### Psychological distress

Distress was measured by four items (e.g., *Have you recently been feeling unhappy and depressed*; α = 0.84) selected from the General Health Questionnaire (GHQ-12 [46–47]), which is a tool designed to screen for mental health problems in the general population. These four items represented the anxiety/depression factor proposed by Graetz [48]. It has been suggested that Graetz’s three-factor structure is the most preferable model for the GHQ-12 [49]. Responses were rated on a 4-point scale ranging from 1 = *not at all* to 4 = *much more than usual*. The mean scores of the responses were computed.

#### Sleep problems

Sleep problems (α = 0.82) were assessed with the 4-item Jenkins Sleep Questionnaire (JSQ [50]). The participants were asked to rate how often during the past few weeks they experienced difficulty falling asleep, waking up feeling tired and worn out after the usual amount of sleep, difficulty staying asleep (including waking up too early), and waking up several times per night. The items were judged on a scale of 1 (*not at all*) to 6 (*every night*). Sleep problems were calculated as a mean of the responses.

### Statistical analysis

Differences between native and foreign-born nurses in the hypothesized associations were tested with multiple linear regression (crude models). First, the interaction effect was examined in the sample of 956 participants. Then, in order to reduce the risk of making type I or II error due to substantially unequal sample sizes (*n* = 744 native nurses vs. *n* = 212 foreign-born nurses), we drew five random samples of 212 observations from the native Finnish nurses’ group to have even number of participants in the native nurses’ group and the foreign-born nurses’ group. Thereafter, we imputed missing values and reran the moderation analysis for each randomly drawn sample separately.

The associations of cross-cultural competence with perceived time pressure, distress, and sleep problems were first examined using multiple linear regression (separate analysis for each outcome). The analysis was conducted in three steps. First, the effect of overall cross-cultural competence on the outcomes was examined (Model 1). In the second step, the associations of four dimensions of cross-cultural competence with the outcomes were tested in separate models for each dimension (Model 2). In the final model (Model 3), the effects of four dimensions of cross-cultural competence on the outcomes were examined simultaneously in the same model. Cross-cultural competence (including its four dimensions) and the outcome variables were treated as continuous variables. All these models were adjusted for gender, age, employment sector, and frequency of interacting with patients and colleagues from different cultures (age was treated as a continuous variable, while the other control variables—as categorical).

The final multiple linear regression models were tested in SEM in order to correct for measurement errors. Goodness-of-fit of the SEM models was evaluated based on: the chi-square test (χ^2^), the root mean squared error of approximation (RMSEA), the comparative fit index (CFI), and the standardized root mean squared residual (SRMR). A non-significant chi-square value indicates that the model is a good fit to the data [51]. This index has been shown, however, to be highly sensitive to sample size, especially when the number of observations exceeds 200 [52]. RMSEA values of 0.05 and less than 0.08 indicate a good and a reasonable fit, respectively. For CFI, values above 0.90 and 0.95 represent an acceptable and a good fit, respectively. The recommended value for SRMR is less than 0.08 [51].

Older nurses were underrepresented in the study sample (the mean age of our participants was 36.19, whereas of the entire population of registered nurses in Finland below 63 years of age—43.72; data from the Central Register). To reduce the risk of biased results, we adjusted our data to reflect age groups in the population of registered nurses aged 62 or below by using post-stratification weighting (sampling weights with the full information maximum likelihood estimation) for the obtained SEM models.

The differences between native and foreign-born nurses with respect of descriptive characteristics were examined with the *t* test, the chi square test (χ^2^), and the Mann-Whitney *U* test. The statistical analysis was performed using Stata/SE 14 software (except for post-stratification weighting conducted in R statistical software).

## Results

Table 1 summarizes the characteristics of the study participants stratified by nativity status. Foreign-born nurses were older than native Finnish nurses, but the groups did not differ in the proportion of women and men. Foreign-born nurses scored higher on cross-cultural empathy, skills, and motivation, but lower on cross-cultural positive attitudes compared with native nurses. Finnish nurses reported higher levels of perceived time pressure than foreign-born nurses. There were no differences between the groups in levels of distress or sleep problems.

**Table 1:** The Characteristics of the original study sample by nativity status.

	Native nurses		Foreign-born nurses		
Study variables	*n* (%)	*M* (*SD*)	*n* (%)	*M* (*SD*)	*p*-value for a difference
Gender	743		212		.118
Women	676 (91.0)		200 (94.3)		
Men	67 (9.0)		12 (5.7)		
Age	744	34.52 (8.89)	212	42.03 (9.50)	< .001
Employment sector	732		201		< .001
Municipal (primary care)	597 (81.6)		109 (54.2)		
State (hospitals)	21 (2.9)		34 (16.9)		
Private and others*	114 (15.6)		58 (28.9)		
Interacting with patients from different cultures	735		207		.554
Rarely/not at all	222 (30.2)		78 (37.7)		
Monthly	182 (24.8)		31 (15.0)		
Daily/weekly	331 (45.0)		98 (47.3)		
Interacting with colleagues from different cultures	694		206		< .001
Rarely/not at all	263 (37.9)		30 (14.6)		
Monthly	73 (10.5)		13 (6.3)		
Daily/weekly	358 (51.6)		163 (79.1)		
Overall cultural competence	704	3.68 (0.52)	190	3.73 (0.49)	.287
Cross-cultural competence subscales					
Empathy	731	3.81 (0.76)	200	3.99 (0.81)	.003
Skills	727	4.00 (0.62)	199	4.25 (0.65)	< .001
Positive attitudes	736	2.65 (0.74)	206	2.02 (0.76)	< .001
Motivation	729	3.92 (0.65)	204	4.10 (0.67)	.001
Perceived time pressure	731	3.69 (1.08)	207	3.47 (1.16)	.010
Psychological distress	736	1.92 (0.71)	209	1.99 (0.73)	.281
Sleep problems	731	2.49 (1.10)	209	2.43 (1.24)	.526

*M* = mean; *n* = number of participants; *SD* = standard deviation.

* Other employment sectors include universities and other research institutions, medical industry, governmental offices, etc.

We found that nativity status moderated the association between cross-cultural skills and distress (*p* = .025; cross-over interaction: the direction of the association was negative in native nurses and positive—in foreign-born nurses) in the unadjusted analysis performed on the sample of 956 participants. There was, however, no moderating effect of nativity status on the associations of cross-cultural competence, empathy, positive attitudes, and motivation with perceived time pressure, distress, or sleep problems (all *p*s > .05). The differences between native and foreign-born nurses in the hypothesized associations were non-significant (all *p*s > .05; unadjusted models) when comparing foreign-born nurses’ group with each of the five randomly drawn samples of 212 Finnish nurses (except for the observed difference in the associations between positive attitudes and time pressure (*p* = .035), skills and distress (*p* = .027), as well as empathy and sleep problems (*p* = .044) in one out of five models). Due to insufficient evidence for the moderating effect of nativity status, further analysis was conducted with native and foreign-born nurses combined.

The results of the multiple linear regression analysis adjusted for all the control variables are presented in Table 2. Models 1 showed that overall cross-cultural competence was negatively associated with perceived time pressure, distress, and sleep problems. In models 2, we observed significant negative associations of cross-cultural empathy, skills, positive attitudes, and motivation with perceived time pressure and sleep problems, and negative associations of empathy and skills with distress. In Models 3, in which all dimensions were entered simultaneously, only the associations of cross-cultural empathy with perceived time pressure and distress remained significant. To ensure that the attenuation of most associations observed in Models 2 was not due to multicollinearity between the dimensions of cross-cultural competence, we used the variance inflation factor (VIF). The results showed that multicollinearity was not present in the data (VIF range 1.03–1.55, *M* = 1.22). Thus, Models 3 were selected as the final models.

**Table 2:** The results of the multiple linear regression examining associations of cross-cultural competence with perceived time pressure, psychological distress, and sleep problems (n = 956).

Predictors	β	*b*	95% CI	*p*-value	Adjusted *R*^*2*^
Perceived time pressure					
Model 1					4.7
Overall cross-cultural competence	–0.11	–0.24	[–0.38, –0.11]	< .001	
Model 2					
Empathy	–0.13	–0.18	[–0.27, –0.09]	< .001	5.0
Skills	–0.08	–0.13	[–0.24, –0.02]	.017	4.0
Positive attitudes	–0.07	–0.09	[–0.18, –0.00]	.041	3.8
Motivation	–0.07	–0.11	[–0.22, –0.00]	.044	3.8
Model 3					4.8
Empathy	–0.11	–0.15	[–0.26, –0.05]	.004	
Skills	–0.03	–0.05	[–0.18, 0.08]	.414	
Positive attitudes	–0.03	–0.04	[–0.13, 0.06]	.469	
Motivation	0.00	0.00	[–0.13, 0.13]	.952	
Psychological distress					
Model 1					1.4
Overall cross-cultural competence	–0.12	–0.16	[–0.25, –0.08]	< .001	
Model 2					
Empathy	–0.19	–0.17	[–0.23, –0.11]	< .001	3.5
Skills	–0.07	–0.08	[–0.15, –0.00]	.038	0.5
Positive attitudes	–0.06	–0.06	[–0.12, 0.00]	.066	0.4
Motivation	–0.04	–0.05	[–0.12, 0.03]	.208	0.2
Model 3					3.3
Empathy	–0.20	–0.18	[–0.25, –0.11]	< .001	
Skills	–0.01	–0.02	[–0.10, 0.07]	.714	
Positive attitudes	–0.01	–0.01	[–0.07, 0.05]	.776	
Motivation	0.05	0.05	[–0.03, 0.14]	.216	
Sleep problems					
Model 1					2.2
Overall cross-cultural competence	–0.12	–0.27	[–0.41, –0.13]	< .001	
Model 2					
Empathy	–0.11	–0.16	[–0.26, –0.07]	.001	1.9
Skills	–0.09	–0.17	[–0.28, –0.05]	.004	1.6
Positive attitudes	–0.08	–0.12	[–0.21, –0.03]	.012	1.3
Motivation	–0.08	–0.14	[–0.25, –0.03]	.014	1.3
Model 3					2.1
Empathy	–0.07	–0.11	[–0.21, 0.00]	.056	
Skills	–0.05	–0.09	[–0.23, 0.04]	.182	
Positive attitudes	–0.05	–0.06	[–0.17, 0.04]	.211	
Motivation	–0.01	–0.02	[–0.15, 0.12]	.788	

Model 2: each dimension of cross-cultural competence in a separate model. Model 3: all dimensions of cross-cultural competence in the same model. All models adjusted for gender, age, employment sector, and frequency of interacting with patients and colleagues from different cultures. The significance levels presented here are for the unstandardized regression coefficients. *b* = unstandardized regression coefficient; β = standardized regression coefficient; CI = confidence interval; adjusted *R*^*2*^ = fraction of explained variance (%) adjusted for the number of predictors in the model.

**Fig 1:**
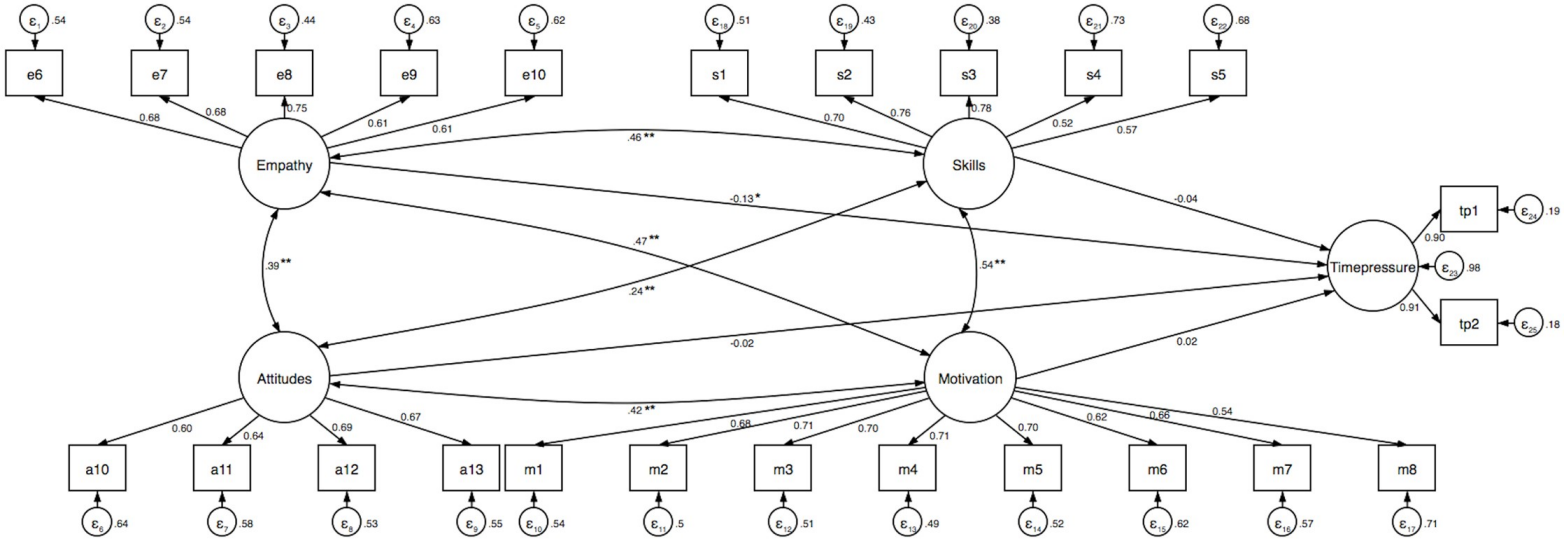
Structural equation model examining the association between cross-cultural competence and perceived time pressure. Standardized coefficients are reported and the significance levels of paths and covariates presented here are for the standardized regression coefficients. Model adjusted for gender, age, employment sector, and frequency of interacting with patients and colleagues from different cultures. Goodness-of-fit indices: χ^2^ (243) = 1092.38, *p* < .001, RMSEA = 0.06, CFI = 0.89, SRMR = 0.06 (index obtained from the estimation of goodness-of-fit of the model tested with the maximum likelihood, *n* = 820). *R*^2^_time pressure_ = 2.4%. * *p* < .01. ** *p* < .001.

**Fig 2:**
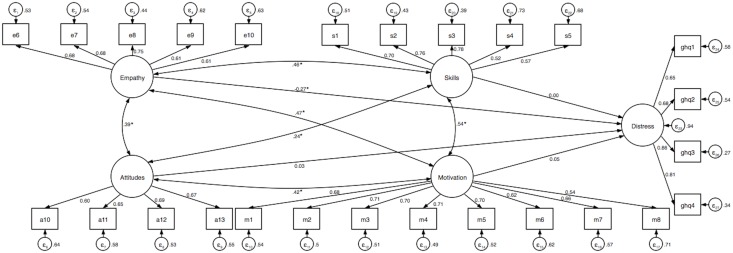
Structural equation model examining the association between cross-cultural competence and psychological distress. Standardized coefficients are reported and the significance levels of paths and covariates presented here are for the standardized regression coefficients. Model adjusted for gender, age, employment sector, and frequency of interacting with patients and colleagues from different cultures. Goodness-of-fit indices: χ^2^ (289) = 1168.29, *p* < .001, RMSEA = 0.06, CFI = 0.89, SRMR = 0.05 (index obtained from the estimation of goodness-of-fit of the model tested with the maximum likelihood, *n* = 819). *R*^2^_distress_ = 5.6%. * *p* < .001.

**Fig 3:**
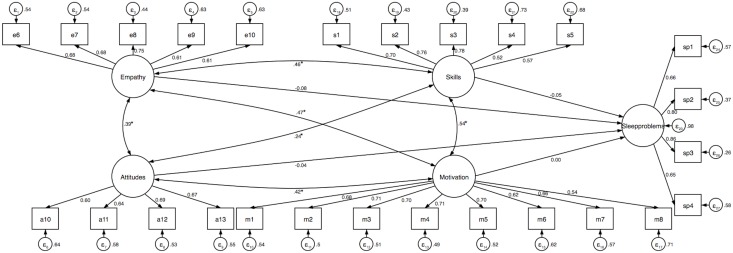
Structural equation model examining the association between cross-cultural competence and sleep problems. Standardized coefficients are reported and the significance levels of paths and covariates presented here are for the standardized regression coefficients. Model adjusted for gender, age, employment sector, and frequency of interacting with patients and colleagues from different cultures. Goodness-of-fit indices: χ^2^ (289) = 1167.68, *p* < .001, RMSEA = 0.06, CFI = 0.89, SRMR = 0.05 (index obtained from the estimation of goodness-of-fit of the model tested with the maximum likelihood, *n* = 816). *R*^2^_sleep problems_ = 1.8%. * *p* < .001.

To adjust for measurement errors, the final models of the multiple linear regression were tested in SEM. The results of the analysis are presented in Table 3 and Figs 1–3. We found that of all four dimensions of cross-cultural competence, only empathy was associated with perceived time pressure and distress when controlling for gender, age, employment sector, and frequency of interacting with patients and colleagues from different cultures. None of the dimensions of cross-cultural competence was associated with sleep problems. The three models provided an acceptable fit to the data.

**Table 3:** The results of the structural equation modeling examining associations of cross-cultural competence with perceived time pressure, psychological distress, and sleep problems (n = 877).

Predictors	*b*	95% CI	*p*-value
Perceived time pressure			
Empathy	–0.21	[–0.37, –0.05]	.010
Skills	–0.06	[–0.22, 0.09]	.424
Positive attitudes	–0.03	[–0.16, 0.10]	.641
Motivation	.04	[–0.17, 0.26]	.702
Psychological distress			
Empathy	–0.23	[–0.32, –0.14]	< .001
Skills	0.00	[–0.08, 0.09]	.978
Positive attitudes	0.02	[–0.05, 0.10]	.513
Motivation	0.06	[–0.06, 0.18]	.319
Sleep problems			
Empathy	–0.10	[–0.23, 0.03]	.123
Skills	–0.06	[–0.19, 0.06]	.319
Positive attitudes	–0.04	[–0.14, 0.06]	.457
Motivation	0.01	[–0.16, 0.18]	.951

All models adjusted for gender, age, employment sector, and frequency of interacting with patients and colleagues from different cultures. *b* = unstandardized regression coefficient; CI = confidence interval.

When post-stratification weighting was applied to the above SEM models, we found that cross-cultural empathy was associated with all outcome variables (perceived time pressure: β = –0.13, *b* = –0.18, 95% CI [–0.33, –0.03], *p* = .018; distress: β = –0.23, *b* = –0.19, 95% CI [–0.27, –0.11], *p* < .001; sleep problems: β = –0.14, *b* = –0.17, 95% CI [–0.29, –0.06], *p* = .004). We did not observe the associations of the remaining cross-cultural competence dimensions with any indicator of well-being (all *p*s > .05). The three models provided an acceptable fit to the data (perceived time pressure: χ^2^ (242) = 1324.59, *p* < .001, RMSEA = 0.07, CFI = 0.87, SRMR = 0.06, *R*^2^_time pressure_ = 2.9%; distress: χ^2^ (289) = 1449.69, *p* < .001, RMSEA = 0.07, CFI = 0.87, SRMR = 0.06, *R*^2^_distress_ = 4.9%; sleep problems: χ^2^ (289) = 1425.29, *p* < .001, RMSEA = 0.07, CFI = 0.87, SRMR = 0.05, *R*^2^_sleep problems_ = 3.2%).

## Discussion

The aim of the present study was to examine whether cross-cultural competence (empathy, skills, positive attitudes, and motivation) is associated with perceived time pressure, distress, and sleep problems in registered nurses, and whether native and foreign-born nurses may differ in these potential associations. According to our results, of all four dimensions of cross-cultural competence, only cross-cultural empathy may be a protective factor against perceived time pressure, distress, and sleep problems independently of gender, age, employment sector, and frequency of interacting with patients and colleagues from different cultures. Native and foreign-born nurses are unlikely to differ in these observed associations.

Our results are consistent with recent reports showing that culturally sensitive nurses are at lower risk of perceived distress [53] and that higher empathy is associated with well-being among nurses in Canada [54], a country known as a multicultural society. The present findings are also in agreement with a study by Ulrey & Amason [33] which showed that cultural sensitivity and empathy may benefit health care providers by reducing their levels of stress and anxiety. Differences between native and foreign-born health care professionals in the associations between cross-cultural competence and well-being indicators have not been examined in these previous investigations [33, 53–54]. The results of our research are not consistent with those of a U.S. study [14] which revealed that cross-cultural competence is a predictor of job satisfaction among nurses. The fact that this study was mostly focused on employees’ perceptions of organizational-level aspects of cross-cultural competence could possibly contribute to this inconsistency. Our results are also not in line with a previous Korean study [15] which indicated that of all measured dimensions of cross-cultural competence (cultural knowledge and skill, cultural attitudes, and cultural awareness), positive attitudes are most strongly associated with a decreased risk of burnout among nursing personnel. Cross-cultural differences in emotion suppression could potentially account for this observed discrepancy. A recent Dutch study [55] found that people with a collectivistic background may have a greater tendency to suppress both positive and negative feelings during their everyday interactions compared with people with an individualistic background. This is in line with the idea that people from collectivistic cultures may be especially motivated to pursue harmony when interacting with others, while people from individualistic cultures may be particularly motivated to express (positive) feelings for the maintenance of well-being [56]. The Dutch study also provided further evidence for the notion that the longer people with a collectivistic background live in an individualistic society, the less they suppress their emotions. These findings could explain why empathy may play an important role in the well-being of native and foreign-born nurses working in Finland, while positive attitudes—in the well-being of Korean nursing personnel.

The results revealed that of all four dimensions of cross-cultural competence, only empathy was associated with perceived time pressure, distress, and sleep problems among nursing personnel. Our previous study [35] also highlighted the importance of cross-cultural empathy among health care professionals. Being culturally empathetic may improve intercultural communication with patients and colleagues [32] by encouraging to consider their point of view. This, in turn, may lead to less difficult cross-cultural encounters in the workplace and, consequently, to better well-being among nurses. For example, it has been shown that health care professionals who express empathy have fewer malpractice complaints from their patients [57] and increased well-being [54, 58] and professional satisfaction [59]. Cultural empathy may also contribute to intercultural relationship building [32]. Satisfactory social connections have repeatedly been shown to promote health and prevent illness (see a review [60–61]). According to Hojat [62], empathetic connections between health care providers and patients can be a source of social support for health care professionals that can protect against poorer health-related outcomes.

Contrary to our hypothesis, there were no differences between native and foreign-born nurses in the observed associations of cross-cultural empathy with perceived time pressure, distress, or sleep problems. This suggests that cross-cultural empathy may be as crucial for well-being of native nurses as for that of foreign-born nurses. The results could also reflect the above-described tendency of people with an individualistic background (who constituted the great majority in our study sample) and people with a collectivistic background living in an individualistic society to express their positive feelings [55], which may account for the important role of empathetic/emotional component of cross-cultural competence in the well-being of nurses working in Finland.

Although nativity status did not moderate the observed associations between cross-cultural competence and well-being, our results showed that Finnish nurses reported lower levels of cross-cultural empathy, skills, and motivation than foreign-born nurses. A lack of long history of immigration to Finland could explain why native Finnish nurses scored lower on the three dimensions of cross-cultural competence compared with immigrant nurses. Finland has become a destination for large-scale international migration relatively recently; the immigration to the country started to increase substantially at the beginning of the century [3]. It has been suggested that, with regard to the work-related immigration of health care professionals, such factors as Finland’s geographic location, language difficulties, and slow licensing process of staff trained abroad could hinder the inflow of migrant workers to the country [63]. The fact that Finland remained relatively isolated from international immigration for a long time means that native Finns have had less opportunities to interact with different cultures, in other words, less occasions to develop some components of cross-cultural competence. In contrast, people of foreign origins due to their minority background have more cross-cultural encounters, thus more chances to practice their cross-cultural competence. Indeed, previous studies [37, 64] have shown that more frequent contacts with people from different cultures are associated with higher levels of cross-cultural competence.

Despite lower scores on most dimensions of cross-cultural competence, native nurses reported more positive cross-cultural attitudes than foreign-born nurses. This finding may simply reflect the fact that Finns in general seem to exhibit more positive attitudes towards immigrants compared to citizens of other European countries, including Estonia, which is the main source country for foreign-born nurses in Finland (approximately 45% [65]). Moreover, native nurses were substantially younger than foreign-born nurses in our study sample (34.52 vs. 42.03, *p* < .001), which could also account for the observed difference. Younger people have previously been shown to be more open to ethnic diversity and to have more positive attitudes towards immigrants compared with older generations [66].

The strength of our study was the inclusion of a relatively large, random sample of native Finnish nurses drawn from the entire population of registered nurses in Finland and a total population of migrant nurses working in Finland. Moreover, the use of well-validated instruments measuring cross-cultural competence and well-being increased the validity of the presented results. Finally, to reduce the possibility of biased results due to missing values among those nurses who responded to the questionnaire (incomplete response bias), the multiple imputation approach and the full information maximum likelihood estimation were applied.

There are limitations of our present study that need to be taken into account when interpreting the results. First, the cross-sectional nature of the study does not allow for any conclusions about the causality or direction of the associations under investigation. Thus, it is equally likely that perceived time pressure, distress, and sleep problems may leave less room for empathy among nurses. A bidirectional association is also possible. This issue needs to be further examined in future experimental and longitudinal research. Second, our study was based on self-report measures, which may have introduced reporting and common method bias associated with inflation or attenuation of relationships. We tried to minimize reporting bias by assuring the respondents that their anonymity would remain protected at every stage of the research. To reduce common method bias, the study included only instruments with a good level of reliability (α > = .74). Third, despite the fact that our statistical analysis was conducted while adjusting for the effect of background factors, including gender, age, employment sector, and frequency of interacting with patients and colleagues from different cultures, the possibility of residual confounding cannot be excluded. For example, it is likely that cross-cultural empathy can be a direct manifestation of personality and coping responses, both known to play a role in influencing mental and physical health [67], rather than a separate construct. Therefore, future studies could control for the potential confounding effect of personality traits and coping style on the association between cross-cultural empathy and well-being. Fourth, the sample of foreign-born nurses was not entirely representative of migrant nursing personnel practicing in Finland. To ensure that this sample would not include native Finnish nurses who had obtained their education abroad, those whose mother tongue was either Finnish or Swedish were excluded. We used a mother tongue as a criterion since information on a citizenship or a country of birth was unavailable from the Central Register when selecting the sample. As a result, the foreign-born nurses’ sample did not include nursing professionals born in Sweden who, nevertheless, have been reported to constitute a relatively small proportion of the migrant nurses in Finland (7.5% [68]). Fifth, in the final models, the values of *R*^2^ for the outcomes were low (2.9%–4.9%). This means that our predictors explained only a small proportion of variance in the outcome variables. However, as it has previously been argued, small proportion of variance explained does not negate the statistical significance of the findings and it may still have important practical implications [69]. Finally, the response rate to the questionnaire was relatively low (43.6% and 46.8%), thus our results may have been affected by non-response bias. It is possible that nurses who did not respond were either not able to do this due to, for example, perceived time pressure at work, or not interested in participating because this did not concern them. The risk of the occurrence of such biases was, however, low in the native nurses’ group given that a response rate of 41.0% has been shown to be sufficient for a sample size above 1000 participants [70]. Moreover, the use of post-stratification weighting enabled us to assess whether our results were likely to be biased by underrepresentation of older nurses in our study sample.

## Conclusions

The results of the present study suggest that cross-cultural empathy may protect against perceived time pressure at work, distress, and sleep problems among both native and foreign-born nurses who face cultural diversity in health care settings. Given this, it may be important to help nursing personnel to develop cross-cultural empathy to more efficiently manage cultural differences in the workplace. There is evidence supporting the effectiveness of cross-cultural training on the improvement of cross-cultural competence [36, 71–72], including empathy [72], among health care providers. Therefore, offering this form of training to nurses may be of value. We also suggest that further research is needed to explore other means by which cross-cultural empathy in nursing staff could be increased, for example, acceptance- and mindfulness-based interventions, which have been shown to facilitate culturally responsive mental health care [73]; the latter has also been shown to improve levels of empathy among health care professionals [74–75].

## Supporting information

S1 Dataset (XLS)

S1 Appendix Study questionnaire in Finnish. (PDF)

S2 Appendix Study questionnaire in English. (PDF)

S1 Table (DOCX)
