# Supplementary material for: The association between cross-cultural competence and well-being among registered native and foreign-born nurses in Finland
Source: PLoS One. 2018 Dec 7;13(12):e0208761. doi: 10.1371/journal.pone.0208761 (PMC6285347; doi:10.1371/journal.pone.0208761)
Supplement: S1 Appendix — (PDF) [file pone.0208761.s002.pdf]

TERVEYDEN JA
HYVINVOINNIN LAITOS

Monikulttuurinen hoitajan työ

TAUSTATIEDOT

2. Sukupuoli

☐ Mies
☐ Nainen

3. Ikä: ____

4. Äidinkielesi

☐ suomi
☐ ruotsi
☐ viro
☐ venäjä
☐ saksa
☐ muu, mikä? ____________________

5. Mikä on siviilisäätysi?

☐ naimaton
☐ naimisissa tai avoliitossa tai rekisteröidyssä parisuhteessa
☐ eronnut tai asumuserossa
☐ leski

6. Missä maassa olet syntynyt? ____________________

7. Onko sinulla Suomen kansalaisuus?

☐ kyllä
☐ ei

8. Jos olet avio- tai avoliitossa tai rekisteröidyssä parisuhteessa, mikä on puolisosi/ avopuolisosi äidinkieli?

☐ suomi
☐ ruotsi
☐ viro
☐ venäjä
☐ saksa
☐ muu, mikä? ______________________________

9. Onko taloudessanne kotona asuvia lapsia?

☐ ei
☐ kyllä, montako? ______________________________

TUTKINTOTIEDOT

10. Minä vuonna suoritit sairaanhoitajan/ terveydenhoitajan/ kätilön tutkintosi (merkitse valmistumisvuosi)?

|_|_|_|_|

11. Onko sinulla myös perushoitajan tai lähihoitajan tutkinto?

☐ ei
☐ kyllä

12. Onko sinulla myös joku muu hoitotyön tutkinto tai joku maisteritutkinto (esim. terveystieteiden maisteri)?

☐ ei
☐ kyllä, mikä? ______________________________

PÄÄTOIMEA KOSKEVAT TIEDOT

Vastaa seuraaviin kysymyksiin päätoimesi mukaan. Mikäli et ole tällä hetkellä työssä, vastaa kysymyksiin viimeisimmän työsi mukaan.

13. Nykyinen työllisyystilanteesi

- ☐ vakituinen kokopäivätyö
- ☐ määräaikainen kokopäivätyö
- ☐ vakituinen osa-aikatyö
- ☐ määräaikainen osa-aikatyö (esim. osa-aikalisä)
- ☐ keikkatyö tai vuokratyö
- ☐ kotona tilapäisesti (esim. äitiys-, isyys-, hoito- tai vanhempainvapaa tai vuorotteluvapaa)
- ☐ päätoiminen opiskelija
- ☐ työtön
- ☐ yksityisyrittäjä
- ☐ eläkkeellä
- ☐ muu

14. Nykyinen työvuoromuotosi

- ☐ päivätyö
- ☐ kaksivuorotyö
- ☐ kolmivuorotyö
- ☐ muu, mikä? ____

15. Nykyisen palvelusuhteesi kesto (jos olet keikkatyössä, merkitse viimeisimmän työnantajan palveluksessa yhteensä työskentelemäsi aika)

- ☐ alle vuosi
- ☐ 1-2 vuotta
- ☐ 3-5 vuotta
- ☐ 6-10 vuotta
- ☐ yli 10 vuotta

16. Kuinka paljon teet keskimäärin normaalin työajan lisäksi lisä/ylityötä kolmen viikon jaksossa? Ilmoita vastauksesi tunteina.

____

17. Nykyinen virkanimikkeesi tai työtehtäväsi?

- [ ] sairaanhoitaja
- [ ] terveydenhoitaja
- [ ] kätilö
- [ ] ensihoitaja
- [ ] lähihoitaja/ perushoitaja tai vastaava
- [ ] laitosapulainen tai vastaava
- [ ] osastonhoitaja/ apulaisosastonhoitaja
- [ ] ylihoitaja/ johtava ylihoitaja tai vastaava
- [ ] hallintoylihoitaja
- [ ] kliinisen hoitotyön asiantuntija
- [ ] hoitotyön opettaja
- [ ] järjestöasiantuntija
- [ ] muu sosiaali- tai terveysalan virkanimike/ työtehtävä
- [ ] muu kuin sosiaali- tai terveysalan virkanimike/ työtehtävä

18. Päätoimen työnantajasektori

- [ ] kunnallinen työnantaja (kuten sairaanhoitopiiri tai kunta)
- [ ] valtio työnantajana
- [ ] yksityinen työnantaja
- [ ] yliopisto, muu oppilaitos
- [ ] kolmas sektori
- [ ] en ole tällä hetkellä potilastyössä

19. Päätoimen toimipaikka/ työpaikka/työyksikkö/ toimintaympäristö

- [ ] yliopistollinen keskussairaala
- [ ] keskussairaala
- [ ] muu julkinen sairaala (aluesairaala, kaupungin sairaala)
- [ ] terveyskeskus/ hyvinvointikeskus
- [ ] yksityinen lääkäriasema, -keskus tai sairaala
- [ ] säätiö, yhdistys tai järjestö
- [ ] valtion virasto, laitos tai sairaala
- [ ] sosiaalihuolto (palvelutalot, tehostetun hoidon yksiköt, sosiaalihuollon avopalvelut)
- [ ] muu

20. Jos valitsit vaihtoehdon, "muu" kirjoita alla olevaan tekstikenttään päätoimen työpaikkasi.

____

21. Potilastyötä tehdessäsi työskenteletkö pääsääntöisesti (jos et ole potilastyössä, siirry seuraavaan kysymykseen)

- [ ] avohuollossa, poliklinikalla tai vastaanotolla
- [ ] ensihoidossa
- [ ] hallinnossa
- [ ] kotihoidossa
- [ ] kotisairaalassa
- [ ] leikkaussalissa
- [ ] liikkuvassa palveluyksikössä
- [ ] neuvolassa, koulu- tai opiskeluterveydenhuollossa
- [ ] palvelutalossa
- [ ] päivystyksessä (päivystysosasto)
- [ ] synnytyssalissa
- [ ] teho- ja valvontaosastolla
- [ ] tutkimusyksikössä (esim. laboratorio, kuvantamispalvelut, endoskopia, sydänasema)
- [ ] vuodeosastolla tai ympärivuorokautisen hoivan yksikössä
- [ ] muu

22. Päätoimen sijainti

- [ ] suuri kaupunki (yli 100 000 asukasta)
- [ ] keskisuuri kaupunki tai kunta (20 000-100 000 asukasta)
- [ ] pieni taajama tai kunta (alle 20 000 asukasta)

23. Oletko esimiestehtävissä

- [ ] en
- [ ] kyllä

24. Oletko tehnyt sairaanhoitajaksi/terveydenhoitajaksi/kätilöksi valmistumisen jälkeen muuta kuin tutkintoasi vastaavaa työtä Suomessa?

- [ ] en
- [ ] kyllä, lähihoitajan/ perushoitajan työtä
- [ ] kyllä, muuta sosiaali- tai terveysalan työtä
- [ ] kyllä, muuta kuin sosiaali- ja terveysalan työtä

25. Oletko ollut työttömänä viimeisen 12 kuukauden aikana?

- [ ] kyllä, yhteensä kuinka monta kuukautta? ________________________________
- [ ] en

26. Jos vastasit edelliseen kysymykseen kyllä, niin mikä seuraavista kuvaa parhaiten tilannettasi?

- ☐ olen hakenut töitä, mutta minua ei ole valittu/ työtä ei ole tarjolla
- ☐ en ole hakenut töitä, koska työ ei sovi tämänhetkiseen elämäntilanteeseeni
- ☐ muu, mikä? ______________________________

MONIKULTTUURISUUS JA KULTTUURINEN OSAAMINEN

27. Oletko saanut koulutusta liittyen monikulttuurisuuteen?

- ☐ en
- ☐ kyllä, osana tutkintoani
- ☐ kyllä, tutkinnon jälkeen, esim. työpaikka- tai täydennyskoulutus tms.
- ☐ kyllä, olen osallistunut monikulttuurisuuteen liittyvään kehittämistyöhön tai projektiin

28. Kuinka usein keskimäärin kohtaat eri kulttuureista kotoisin olevia potilaita omassa työssäsi?

- ☐ en lainkaan
- ☐ päivittäin
- ☐ viikoittain
- ☐ kuukausittain
- ☐ harvemmin

29. Onko työyhteisössäsi/ työyksikössäsi eri kulttuureista kotoisin olevia työtovereita?

- ☐ ei ollenkaan
- ☐ eri kulttuureista kotoisin olevia on selkeästi vähemmän kuin kantasuomalaisia
- ☐ eri kulttuureista kotoisin olevia on yhtä paljon tai lähes yhtä paljon kuin kantasuomalaisia
- ☐ eri kulttuureista kotoisin olevia on selkeästi enemmän kuin kantasuomalaisia

30. Kuinka usein keskimäärin olet työssäsi tekemisissä eri kulttuureista kotoisin olevien työtovereidesi kanssa?

- ☐ en lainkaan
- ☐ päivittäin
- ☐ viikoittain
- ☐ kuukausittain
- ☐ harvemmin

31. Seuraavassa on monikulttuurisuuteen liittyviä väittämiä. Valitse mielipidettäsi parhaiten kuvaava vaihtoehto kussakin väittämässä.

	täysin eri mieltä	jokseen-kin eri mieltä	ei samaa eikä eri mieltä	jok-seenkin samaa mieltä	täysin samaa mieltä
Minusta on rikkaus, että on eri kulttuuritaustoista tulevia ystäviä.	☐	☐	☐	☐	☐
Kulttuurinen monimuotoisuus on myös rikkaus.	☐	☐	☐	☐	☐
Minusta on innostavaa hoitaa maahanmuuttajataustaisia potilaita.	☐	☐	☐	☐	☐
Minusta työskentely monikulttuurisessa tiimissä on rikkaus.	☐	☐	☐	☐	☐
Juttelen mielelläni maahanmuuttajataustaisten ihmisten kanssa heidän kokemuksistaan täällä Suomessa.	☐	☐	☐	☐	☐
Kanssakäyminen muista kulttuureista tulevien ihmisten kanssa auttaa minua pohtimaan omaa kulttuuritaustaani.	☐	☐	☐	☐	☐
Keskustelemalla maahanmuuttajataustaisten potilaiden kanssa opin tuntemaan erilaisia kulttuureita.	☐	☐	☐	☐	☐
Haluaisin saada valmennusta, neuvontaa ja koulusta parantaakseni ymmärrystäni maahanmuuttajataustaisista potilaista.	☐	☐	☐	☐	☐
Minulle on tärkeää, että potilaiden hoidossa otetaan huomioon heidän kulttuuriset tarpeensa ja henkilökohtaiset arvonsa.	☐	☐	☐	☐	☐
Minusta on rasittavaa, kun kauan sitten Suomeen muuttaneet ihmiset eivät osaa suomea kunnolla.	☐	☐	☐	☐	☐
Maahanmuuttajien tulisi sopeutua suomalaiseen yhteiskuntaan, ei päinvastoin.	☐	☐	☐	☐	☐
Eri toimijat ja suuri yleisö kiinnittävät liikaa huomiota maahanmuuttajien erityistoiveisiin.	☐	☐	☐	☐	☐
Käsitykseni mukaan maahanmuuttajat näkevät usein syrjintää siellä, missä tosiasiassa vain noudatetaan yleisiä sääntöjä.	☐	☐	☐	☐	☐

32. Seuraavassa on monikulttuuriseen asiakas/potilastyöhön liittyviä väittämiä. Valitse mielipidettäsi parhaiten kuvaava vaihtoehto kussakin väittämässä.

	täysin eri mieltä	jokseenkin eri mieltä	ei samaa eikä eri mieltä	jokseenkin samaa mieltä	täysin samaa mieltä
Jos potilas ei ymmärrä hyvin suomea, käytän enemmän aikaa kertoakseni hänelle eri hoitovaihtoehdoista.	☐	☐	☐	☐	☐
Jotta sovittu hoitotavoite saavutetaan, kysyn maahanmuuttajataustaiselta potilaalta, millaista tukea hän tarvitsee.	☐	☐	☐	☐	☐
Jos potilas ei ymmärrä hyvin suomea, käytän enemmän aikaa keskustellakseni hänen kanssaan hänen odotuksistaan ja peloistaan.	☐	☐	☐	☐	☐
Ihmisen kulttuurisidonnaiset ominaisuudet (esim. arvot, käyttäytymisnormit ja uskomukset) vaikuttavat huomattavasti hänen sairauskäsityksiinsä. Siksi terveydenhuollon ammattilaisten pitäisi ottaa ne huomioon.	☐	☐	☐	☐	☐
Otan huomioon potilaan perhearvot, uskonnon ym., jos ne vaikuttavat hoidon kannalta oleellisilta.	☐	☐	☐	☐	☐
Kun olen työssäni tekemisissä maahanmuuttajataustaisten potilaiden kanssa, olen usein epävarma, kiukkuinen ja turhautunut.	☐	☐	☐	☐	☐
Minun on usein vaikea suhtautua potilaan pohdintoihin, jos hänen sosiaalinen ja kulttuurinen taustansa on hyvin erilainen kuin omani.	☐	☐	☐	☐	☐
Turhaudun, jos maahanmuuttajataustainen potilas ei ymmärrä, mitä sanon.	☐	☐	☐	☐	☐
Minusta on vaikea puhua hitaasti ja maallikkokielellä potilaalle, jolla on vaikeuksia ymmärtää ohjeitani.	☐	☐	☐	☐	☐
Hoidan mieluummin potilaita, joilla on sama kulttuuritausta kuin minulla, kuin itselleni vieraalta tuntuvia potilaita.	☐	☐	☐	☐	☐
Maahanmuuttajataustaisen potilaan sairauskäsitykset eivät ole olennaisia hoidon onnistumisen kannalta.	☐	☐	☐	☐	☐
Maahanmuuttajien välillä ei ole juurikaan eroja terveyteen liittyvien mahdollisuuksien ja toisaalta sairausriskien kannalta.	☐	☐	☐	☐	☐
Oma kulttuuritaustani ei vaikuta ammatilliseen näkemykseeni, tekemiini arvioihin tai toimintaani.	☐	☐	☐	☐	☐
Muutto toiseen maahan on mullistava elämäntapahtuma, johon voi liittyä psykososiaalista stressiä ja terveysongelmia.	☐	☐	☐	☐	☐

TYÖ JA TYÖYHTEISÖ

Vastaa seuraaviin väittämiin päätoimesi mukaan. Mikäli et ole tällä hetkellä työssä, vastaa väittämiin viimeisimmän työsi mukaan. Valitse mielipidettäsi parhaiten kuvaava vastausvaihtoehto.

33. Seuraavat väittämät koskevat hoitotyötä.

	täysin eri mieltä	jokseen-kin eri mieltä	ei samaa eikä eri mieltä	jok-seenkin samaa mieltä	täysin samaa mieltä
Kartoitan työssäni laaja-alaisesti potilaan fyysisiä, psyykkisiä ja sosiaalisia tarpeita.	☐	☐	☐	☐	☐
Pystyn hyödyntämään hoitotyön asiantuntemustani moniammatillisessa tiimissä.	☐	☐	☐	☐	☐
Yksikössäni on olemassa selkeät ohjeet tulkin tilaamisesta vieraskieliselle potilaalle.	☐	☐	☐	☐	☐
Osaan neuvoa potilasta sähköisten sosiaali- ja terveyspalvelujen käytössä.	☐	☐	☐	☐	☐
Osaan ohjata potilasta sähköisten palvelujen välityksellä.	☐	☐	☐	☐	☐
Osaan varmistaa, että potilasta koskeva tieto etenee hoito- tai palveluketjussa.	☐	☐	☐	☐	☐
Pystyn työssäni vaikuttamaan siihen, että heikossa asemassa olevat potilaat saavat tarvitsemansa tuen, avun ja palvelut.	☐	☐	☐	☐	☐
Perustelen näkemyksiäni potilaalle näyttöön perustuvalla tiedolla tai tutkimuksella.	☐	☐	☐	☐	☐
Seuraan aktiivisesti sosiaali- ja terveydenhuoltoa ohjaavan lainsäädännön muutoksia ja pohdin niiden vaikutuksia työhöni.	☐	☐	☐	☐	☐
Osaan selvittää potilaan kanssa vaihtoehtoisia tai täydentäviä sosiaali- ja terveydenhuollon palveluja.	☐	☐	☐	☐	☐

34. Seuraavissa kohdissa sinua pyydetään arvioimaan työyksikkösi toimintaa. Ajattele tässä sellaista työyksikköä (osasto, asema/klinikka, pienryhmä), joka on työsi kannalta oleellisin.

	täysin eri mieltä	jokseen-kin eri mieltä	ei samaa eikä eri mieltä	jok-seenkin samaa mieltä	täysin samaa mieltä
Pidämme toisemme ajan tasalla työasioissa	☐	☐	☐	☐	☐
Tietoa todella pyritään jakamaan työyksikön sisällä	☐	☐	☐	☐	☐
Asenteemme on ”Toimimme yhdessä”	☐	☐	☐	☐	☐
Jokainen tuntee tulevansa ymmärretyksi ja olevansa hyväksytty	☐	☐	☐	☐	☐

35. Seuraavat väittämät käsittelevät menettelytapoja työpaikallasi.

	täysin eri mieltä	jokseen-kin eri mieltä	ei samaa eikä eri mieltä	jok-seenkin samaa mieltä	täysin samaa mieltä
Kaikilla on oikeus sanoa mielipiteensä ja kokemuksensa itseään koskevissa asioissa	☐	☐	☐	☐	☐
Tehdyt päätökset ovat työpaikallamme olleet johdonmukaisia	☐	☐	☐	☐	☐
Työpaikallamme päätökset eivät ole puolueellisia	☐	☐	☐	☐	☐

36. Seuraavat väittämät liittyvät avun saamiseen ja antamiseen työpaikallasi.

	erittäin harvoin tai en ollenkaan	melko harvoin	jonkin verran	melko usein	erittäin usein
Saan tarvittaessa tukea ja apua työtovereiltani	☐	☐	☐	☐	☐
Saan tarvittaessa tukea ja apua esimieheltäni	☐	☐	☐	☐	☐
Autan ja tuen työtovereitani heidän tarvitessaan apua	☐	☐	☐	☐	☐

37. Miten hyvin seuraavat väittämät kuvaavat nykyistä työtäsi.

	täysin eri mieltä	jokseen-kin eri mieltä	ei samaa eikä eri mieltä	jok-seenkin samaa mieltä	täysin samaa mieltä
Yleisesti ottaen olen hyvin tyytyväinen työhöni	☐	☐	☐	☐	☐
Työni vaatii, että opin uusia asioita	☐	☐	☐	☐	☐
Työssäni saan tehdä paljon erilaisia asioita	☐	☐	☐	☐	☐
Minulla on mahdollisuus kehittää minulle ominaisia kykyjäni	☐	☐	☐	☐	☐

38. Syrjinnällä tarkoitetaan ihmisten eriarvoista kohtelua (ilman hyväksyttävää syytä) ja huonompaan asemaan asettamista sillä perusteella, että he kuuluvat tiettyyn ryhmään. Oletko kokenut henkilökohtaisesti syrjintää työpaikallasi viimeisten 12 kk:n aikana?

	erittäin harvoin tai ei ollenkaan	melko harvoin	joskus	melko usein	erittäin usein tai jatkuvasti
Esimiehen tai johdon taholta?	☐	☐	☐	☐	☐
Kollegoiden tai työtovereiden taholta?	☐	☐	☐	☐	☐
Potilaiden tai omaisten taholta?	☐	☐	☐	☐	☐

TYÖTYYTYVÄISYYS JA TYÖMOTIVAATIO

39. Seuraavassa esitetään väittämiä, jotka liittyvät siihen millaisena voit nähdä muiden suhtautumisen hoitotyön ammattilaisiin. Valitse kunkin väittämän kohdalla mielipidettäsi parhaiten vastaava vaihtoehto.

	täysin eri mieltä	jokseen-kin eri mieltä	ei samaa eikä eri mieltä	jok-seenkin samaa mieltä	täysin samaa mieltä
Olen ylpeä siitä, että olen hoitotyön ammattilainen	☐	☐	☐	☐	☐
Hoitotyön arvot ovat hyvin lähellä omia arvojani	☐	☐	☐	☐	☐
Voisin suositella läheisille ystävilleni hoitotyön ammattilaiseksi ryhtymistä	☐	☐	☐	☐	☐
Hoitotyön ammattilaiset ovat hyvin arvostettuja yhteiskunnassamme	☐	☐	☐	☐	☐
Minua hävettää kertoa muille, että olen hoitotyön ammattilainen	☐	☐	☐	☐	☐

40. Seuraavat väittämät liittyvät mahdollisiin käsityksiisi itsestäsi hoitotyön ammattilaisena. Minut tuntevat hoitotyön ammattilaiset...

	täysin eri mieltä	jokseen-kin eri mieltä	ei samaa eikä eri mieltä	jok-seenkin samaa mieltä	täysin samaa mieltä
Kunnioittavat tapaani tehdä työtäni	☐	☐	☐	☐	☐
Arvostavat työhön liittyviä ideoitani	☐	☐	☐	☐	☐
Arvostavat panostustani	☐	☐	☐	☐	☐
Arvostavat minua hoitotyön ammattilaisena	☐	☐	☐	☐	☐
Ajattelevat, että minua olisi vaikea korvata	☐	☐	☐	☐	☐

41. Seuraavat väittämät koskevat tuntemuksia ja käsityksiä, joita Sinulle on mahdollisesti syntynyt liittyen hoitotyön ammattilaisena olemiseen.

	täysin eri mieltä	jokseen-kin eri mieltä	ei samaa eikä eri mieltä	jok-seenkin samaa mieltä	täysin samaa mieltä
Kun joku arvostelee hoitotyön ammattilaisia, se tuntuu minusta ikään kuin henkilökohtaiselta loukkaukselta	☐	☐	☐	☐	☐
Kun puhun hoitotyön ammattilaisista, puhun useammin ”meistä” kuin ”heistä”	☐	☐	☐	☐	☐
Olen hyvin kiinnostunut siitä, mitä muut ajattelevat hoitotyön ammattilaisista	☐	☐	☐	☐	☐
Jos tiedotusvälineissä arvostellaan hoitotyön ammattilaisia, tunnen itseni kiusaantuneeksi	☐	☐	☐	☐	☐
Hoitotyön ammattilaisten menestyminen on myös minun menestymistäni	☐	☐	☐	☐	☐
Kun joku kehuu hoitotyön ammattilaisia, se tuntuu minusta ikään kuin henkilökohtaiselta kohteliaisuudelta	☐	☐	☐	☐	☐

42. Työpaikan vaihtoaikeet

	en	mahdollisesti	kyllä
Oletko suunnitellut vaihtavasi työpaikkaa/ työnantajaa?	☐	☐	☐
Oletko suunnitellut lähteväsi työskentelemään johonkin muuhun maahan seuraavan 12 kk aikana?	☐	☐	☐

43. Seuraavaksi kysymme mielipidettäsi erilaisiin väittämiin siitä, miten hyvin esimiehet ja työtoverit ovat tehneet Sinulle selväksi, miten työpaikkanne toimii ja millaisia toimintatapoja työpaikallanne on.

	erittäin huonosti tai ei lainkaan	melko huonosti	melko hyvin	erittäin hyvin
Sinulle on selvitetty, mitä tehtävistä työssänne pääosin vastaat	☐	☐	☐	☐
Sinulle on selvitetty työaikoihin sekä työstä poissaoloihin liittyvät säännöt	☐	☐	☐	☐
Sinulle on kerrottu, kuka tai mikä taho työpaikalla voi auttaa sinua missäkin asiassa	☐	☐	☐	☐
Sinulle on selvitetty, missä asioissa voit kääntyä esimiehesi puoleen	☐	☐	☐	☐
Sinulle on kerrottu, mistä saat tietoa työpaikkaa koskevista päätöksistä ja muutoksista	☐	☐	☐	☐
Sinulle on kerrottu, kenen puoleen voi kääntyä, jos tulee kiusatuksi työpaikalla	☐	☐	☐	☐

44. Kuinka usein kukin alla mainittu asia on selvästi häirinnyt, huolestuttanut tai rasittanut Sinua työssä viimeisen 6 kk:n aikana? Valitse mielipidettäsi parhaiten kuvaava vastausvaihtoehto kussakin kohdassa.

	erittäin harvoin tai ei koskaan	melko harvoin	silloin tällöin	melko usein	erittäin usein tai jatku-vasti
Jatkuva kiire ja tekemättömien töiden paine	☐	☐	☐	☐	☐
Liian vähän aikaa työn tekemiseen kunnolla	☐	☐	☐	☐	☐
Muuttuvat sähköiset tietojärjestelmät	☐	☐	☐	☐	☐
Hankalat, huonosti toimivat tietotekniset laitteet tai ohjelmat	☐	☐	☐	☐	☐
Henkilöstön vaihtuvuus, lyhytaikaiset sijaiset	☐	☐	☐	☐	☐
Potilailla on usein erilaiset odotukset hoidosta kuin henkilökunnalla	☐	☐	☐	☐	☐
Hankalat potilaat, jotka valittavat, syyttelevät, tai arvostelevat	☐	☐	☐	☐	☐
Potilaat eivät halua osallistua hoitoon, ovat passiivisia	☐	☐	☐	☐	☐
Omat riittämättömät ammatilliset tiedot tai taidot	☐	☐	☐	☐	☐
Vastuu potilaista	☐	☐	☐	☐	☐

TERVEYS JA HYVINVOINTI

45. Millainen on terveydentilasi verrattuna muihin ikäisiisi?

- [ ] hyvä
- [ ] melko hyvä
- [ ] keskitasoinen
- [ ] melko huono
- [ ] huono

46. Oletetaan että työkykysi on parhaimmillaan saanut 10 pistettä ja että 0 tarkoittaisi, että et pysty lainkaan työhön. Minkä pistemäärän antaisit nykyiselle työkyvyllesi?

	0	1	2	3	4	5	6	7	8	9	10	
erittäin huono	☐	☐	☐	☐	☐	☐	☐	☐	☐	☐	☐	erittäin hyvä

47. Stressillä tarkoitetaan tilannetta, jossa ihminen tuntee itsensä jännittyneeksi, levottomaksi, hermostuneeksi tai ahdistuneeksi tai hänen on vaikea nukkua asioiden vaivatessa jatkuvasti mieltä. Tunnetko sinä nykyisin tällaista stressiä?

- [ ] en lainkaan
- [ ] vain vähän
- [ ] jonkin verran
- [ ] melko paljon
- [ ] erittäin paljon

48. Seuraavat kysymykset koskevat hyvinvointiasi muutaman viimeksi kuluneen viikon aikana. Valitse kunkin väittämän kohdalla sopivin vaihtoehto.

	en ollenkaan	en enempää kuin tavallisesti	jonkin verran enemmän kuin tavallisesti	paljon enemmän kuin tavallisesti
Oletko viime aikoina valvonut paljon huoliesi takia?	☐	☐	☐	☐
Oletko viime aikoina tuntenut olevasi jatkuvasti ylirasittunut?	☐	☐	☐	☐
Onko sinusta viime aikoina tuntunut ettet voisi selviytyä vaikeuksista?	☐	☐	☐	☐
Oletko viime aikoina tuntenut itsesi onnettomaksi ja masentuneeksi?	☐	☐	☐	☐

49. Kuinka usein Sinulla on viimeksi kuluneiden neljän viikon aikana ollut seuraavia oireita?

	ei lainkaan	1-3 yönä /kk	noin yhtenä yönä/ vko	2-4 yönä /vko	5-6 yönä/ vko	joka yö
Nukahtamisvaikeuksia	☐	☐	☐	☐	☐	☐
Heräilyä useita kertoja yön aikana	☐	☐	☐	☐	☐	☐
Vaikeuksia pysyä unessa (mukaan lukien liian aikaisin heräämisen)	☐	☐	☐	☐	☐	☐
Olet tuntenut itsesi väsyneeksi ja uupuneeksi herättyäsi tavallisen yöunen jälkeen?	☐	☐	☐	☐	☐	☐

50. Alla olevaan tilaan voit kirjoittaa huomioitasi ja mielipiteitäsi tästä tutkimuksesta. Suuri kiitos vastauksistasi!

____

____

____
