# Supplementary material for: The association between cross-cultural competence and well-being among registered native and foreign-born nurses in Finland
Source: PLoS One. 2018 Dec 7;13(12):e0208761. doi: 10.1371/journal.pone.0208761 (PMC6285347; doi:10.1371/journal.pone.0208761)
Supplement: S2 Appendix — (PDF) [file pone.0208761.s003.pdf]

TERVEYDEN JA
HYVINVOINNIN LAITOS

MULTICULTURAL NURSE'S JOB

BACKGROUND INFORMATION

Welcome to this survey of multiculturalism and working conditions. Please consider the questions and statements in this form on the basis of your current situation. If you are currently not at work, base your answers on your last job. Try and answer all the questions in the form. If one of them seems too difficult or you have nothing to base your answers on, skip to the next question. You do not have to answer every question to move on in the survey. You can also take a break by clicking on the “Break” button. Next time, you can carry on from the question where you left off. To send the form, click on the Submit button on the last page.

1. Please begin by writing down the ID number in your covering letter *

ID number

____

4 characters remaining

2. Gender

○ Male

○ Female

3. Age

____

2 characters remaining

4. What is your mother tongue?

◯ Finnish

◯ Swedish

◯ Estonian

◯ Russian

◯ Other, please specify

____

5. What is your marital status?

◯ Single

◯ Married/living together or in a registered partnership

◯ Separated/divorced

◯ Widowed

6. In what country were you born?

____

7. If you are married/living together or in a registered partnership, what is your spouse’s mother tongue?

○ Finnish

○ Swedish

○ Estonian

○ Russian

○ Other, please specify

____

8. Do you have any children living at home with you?

○ No

○ Yes, how many?

____

DEGREE OR QUALIFICATION DETAILS

9. In what country did you complete your qualification/degree as a registered nurse/public health nurse/midwife?

○ I completed the entire qualification/degree in Finland only

○ I completed the entire qualification/degree abroad only, in:

____

○ I completed the entire qualification/degree abroad but had to take supplementary studies in Finland to obtain a licence to practise my profession

○ I had to take the entire qualification/degree again in Finland even though I had already completed the same

qualification/degree abroad

◯ I completed part of the my qualification/degree in Finland and part abroad

10. In what year did you complete your qualification/degree as a registered nurse/public health nurse/midwife (write in the year in which you graduated)?

____

4 characters remaining

11. In what year did you receive your licence to practise your profession in Finland?

____

4 characters remaining

12. Did you find obtaining a licence to practise as a professional...

◯ easy

◯ difficult, why?

____

13. Do you also have a qualification/degree as a practical nurse?

◯ No

◯ Yes

14. Do you also have some other qualification/degree in nursing or a Master’s degree (for example, a Master of Health Science degree)?

○ No

○ Yes, which?

____

DETAILS OF MAIN OCCUPATION

While completing this section, please rate the questions and statements in the survey on the basis of your current main occupation. If you are currently not employed, base your answers on your most recent job.

15. Your current employment situation

○ Permanent full-time job
○ Fixed-term full-time job
○ Permanent part-time job
○ Fixed-term part-time job (e.g. part-time pay supplement)
○ Temporary or hired work
○ Temporarily at home (maternity, paternity, child care or parental leave, job alternation leave, etc.)
○ Full-time student
○ Unemployed
○ Self-employed
○ Retired
○ Other

16. Your current working hours arrangement

○ Day job

○ Two shifts

○ Three shifts

○ Other, please specify:

____

17. The duration of your current employment relationship (if you are a temporary or hired worker, please give the total time for which you have been employed by your most recent employer)

○ Select
○ Less than 1 year
○ 1-2 years
○ 3-5 years
○ 6-10 years
○ Over 10 years

18. How many hours per week do you do additional work or overtime on average during a three-week period? (In hours)

____

19. Your current job title or job description

○ Registered nurse
○ Public health nurse
○ Midwife
○ First responder
○ Practical nurse or similar

◯ Hospital assistant or similar
◯ Head nurse/assistant head nurse
◯ Senior nurse/director of nursing or similar
◯ Administrative director of nursing
◯ Clinical nursing expert
◯ Teacher of nursing
◯ Organisation expert
◯ Other social and health sector job title/role
◯ Other than social and health sector job title/role

20. Employment sector of your main occupation

◯ A municipal employer (for example a hospital district or a municipality)
◯ A central government employer
◯ A private employer
◯ A university or other educational institution
◯ Third sector
◯ I do not work with patients at the moment

21. Branch/workplace/unit/operating environment of main occupation

◯ Select
◯ University central hospital
◯ Central hospital
◯ Other public hospital (regional hospital, city hospital)
◯ Health centre/centre for well-being
◯ A private health centre, medical centre or hospital

◯ A foundation, association or organisation
◯ A central government agency, institution or hospital
◯ Social welfare (assisted living facilities, intensive care units, community-based social welfare services)
◯ Other

22. If you selected “Other”, please type the name of the workplace for your main occupation in the text field below.

____

23. When working with patients, do you mainly work in (if you do not work with patients, skip to the following question)

◯ Outpatient care, a clinic or a surgery
◯ First response duties
◯ Administration
◯ Home care
◯ Home hospital care
◯ Theatre
◯ A mobile service unit
◯ A maternity or child health clinic, school or student health care
◯ An assisted living unit
◯ Emergency care (emergency care ward)
◯ Delivery room
◯ Intensive and step down care unit
◯ A research unit (incl. laboratories, imaging services, endoscopy, cardiology unit)
◯ An inpatient ward or 24-hour-care unit
◯ Other

24. Location of main occupation

◯ Large city (more than 100,000 residents)

◯ Middle-size city or municipality (20,000–100,000 residents)

◯ Small town or municipality (less than 20,000 residents)

25. Do you have managerial duties?

◯ No

◯ Yes

26. After graduating as a registered nurse / public health nurse / midwife, have you done other work in Finland besides work that corresponds to your qualification or degree?

◯ No
◯ Yes, I have worked as a practical nurse or similar
◯ Yes, I have done other work in the social welfare or health care sector
◯ Yes, I have done work outside the social welfare or health care sector

27. In your opinion, has your foreign origin been an advantage or a disadvantage when looking for a job in Finland?

◯ Advantage
◯ Advantage and disadvantage
◯ Disadvantage
◯ No significance

28. Have you been unemployed in the past 12 months?

◯ Yes, for how many months in total?

____

◯ No

29. If you answered Yes to the previous question, which one of the following best describes your situation?

◯ I have applied for jobs but have not been hired / there is no work available

◯ I have not applied for jobs, because working is not suitable for my current situation

◯ Other, please specify:

____

MULTICULTURALISM AND CULTURAL COMPETENCE

30. Have you received multiculturality training?

◯ Select
◯ No
◯ Yes, as part of my qualification/degree
◯ Yes, after graduating, e.g. workplace training, other further training etc.
◯ Yes, I have participated in a project/development work related to multiculturalism

31. How often on average do you meet patients from different cultures in your work?

◯ Not at all

○ Daily
○ Weekly
○ Monthly
○ Less than monthly

32. Do you have co-workers who are from a different cultural background in your unit?

○ None
○ There are substantially less workers with a different cultural background than workers of Finnish origin
○ There is an equal or an almost equal number of persons with a different cultural background and those of Finnish origin
○ There are clearly more workers with a different cultural background than workers of Finnish origin

33. To what extent do you deal with colleagues with a different cultural background in your workplace?

○ Not at all
○ Daily
○ Weekly
○ Monthly
○ Less than monthly

34. Please read the following statements associated with multiculturalism. Do you agree or disagree with the statement?

	Fully disagree	Somewhat disagree	Neither agree nor disagree	Somewhat agree	Fully agree
I consider it an enrichment to have friendships with	○	○	○	○	○

people from different cultural backgrounds.					
Cultural diversity is also an enrichment.	○	○	○	○	○
I find it exciting to treat patients with a migration background.	○	○	○	○	○
I consider working in a cross-cultural team an enrichment.	○	○	○	○	○
I enjoy talking to people who have migrated to Finland about their experiences here.	○	○	○	○	○
The interaction with people from other cultural backgrounds helps me reflect upon my own cultural background.	○	○	○	○	○
By communicating with patients with a migration background, I can learn about different cultural orientations.	○	○	○	○	○
I would like to make use of training, advising and educational offers, in order to improve my understanding of patients with a migration background.	○	○	○	○	○
It is important for me to treat patients according to their cultural needs and individual values.	○	○	○	○	○
I find it an imposition, when people who migrated to Finland a long time ago, cannot speak Finnish properly.	○	○	○	○	○
People who migrated to Finland should adapt to society, not the other way around.	○	○	○	○	○
Institutions and the public pay too much attention to the special wishes of migrants.	○	○	○	○	○

I have the impression that migrants often assume discrimination, when in fact general rules are simply being enforced.	○	○	○	○	○

35. Please read the following statements associated with multicultural customer/patient work. For each statement, select the alternative that best describes your opinion.

	Fully disagree	Somewhat disagree	Neither agree nor disagree	Somewhat agree	Fully agree
With patients who do not understand Finnish very well, I take more time to explain the treatment options to them.	○	○	○	○	○
In order to achieve the agreed treatment goal, I ask patients with a migration background what they need in terms of support.	○	○	○	○	○
With patients who do not understand Finnish very well, I take more time to discuss their expectations and fears.	○	○	○	○	○
Culturally specific factors of people (e.g. values, behavior norms, beliefs) influence their understanding of disease significantly, and should therefore be assessed and taken into consideration by healthcare professionals.	○	○	○	○	○
I consider the values of patients in relation to family, religion, etc., if they seem relevant for the treatment.	○	○	○	○	○
In my professional interaction with patients with a migration background, I often feel unsure, angry and frustrated.	○	○	○	○	○
I often find it difficult to relate to the elaborations of	○	○	○	○	○

my patients, when their socio-cultural background is quite different from my own.					
I get impatient when I cannot make myself understood with patients with a migration background.	○	○	○	○	○
I find it difficult to speak slowly in lay language with people who struggle to understand my instructions.	○	○	○	○	○
I prefer treating patients from my own cultural background, than those who seem foreign to me.	○	○	○	○	○
The disease concepts of patients with a migration background are not relevant for treatment success.	○	○	○	○	○
Within the migrant population, there are hardly any differences in terms of health opportunities and disease risks.	○	○	○	○	○
My professional perception, assessment, and behaviour remain untouched by my cultural imprinting.	○	○	○	○	○
The migration experience is a critical life event and can be accompanied by psychosocial stress and a health burden.	○	○	○	○	○

WORK AND WORK COMMUNITY

While completing this section, please rate the questions and statements in the survey on the basis of your current main occupation. If you are currently not employed, base your answers on your most recent job. For each statement, select the alternative that best describes your opinion.

36. The following statements concern nursing.

	Fully disagree	Somewhat disagree	Neither agree nor disagree	Somewhat agree	Fully agree
In my work, I extensively assess the patient's physical, psychological and social needs.	○	○	○	○	○
I can use my nursing expertise in a multiprofessional team.	○	○	○	○	○
My unit has clear instructions for providing an interpreter for a patient who speaks a foreign language.	○	○	○	○	○
I know how to advise patients in using electronic social and health services.	○	○	○	○	○
I know how to use electronic services to guide patients.	○	○	○	○	○
I know how to make sure that information on a patient moves forward in the care or service chain.	○	○	○	○	○
In my work, I can exert influence to make sure that patients in a vulnerable position have access to the support, assistance and services they need.	○	○	○	○	○
I justify my views to the patient with evidence-based knowledge or research.	○	○	○	○	○
I actively follow changes in the legislation that directs social and health care and consider their impacts on my work.	○	○	○	○	○
I know how to investigate alternative or complementary social and health services with a patient.	○	○	○	○	○

37. The following statements are about the operation of your unit. Think here of the work unit (department, health centre/clinic, team) that is the most essential to your work..

	Fully disagree	Somewhat disagree	Neither agree nor disagree	Somewhat agree	Fully agree
We keep each other informed about work related issues	○	○	○	○	○
There are real attempts to share information throughout the practice	○	○	○	○	○
We have a "we are in it together" attitude	○	○	○	○	○
We feel understood and accepted by each other	○	○	○	○	○

38. The following statements refer to procedures in your workplace.

	Fully disagree	Somewhat disagree	Neither agree nor disagree	Somewhat agree	Fully agree
Everybody is entitled to express an opinion and their experience in matters concerning themselves	○	○	○	○	○
Decisions taken in our workplace have been consistent	○	○	○	○	○
Decisions in our workplace are not biased	○	○	○	○	○

39. The following statements refer to offering and receiving help at your workplace.

	Fully	Somewhat	Neither agree nor	Somewhat	Fully

	disagree	disagree	disagree	agree	agree
I receive support and help from my co-workers when I need it	○	○	○	○	○
I receive support and help from my supervisor when I need it	○	○	○	○	○
I help and support my co-workers when they need it	○	○	○	○	○

40. How well do the following statements describe your job?

	Fully disagree	Somewhat disagree	Neither agree nor disagree	Somewhat agree	Fully agree
Generally speaking, I am very satisfied with my job	○	○	○	○	○
I am required to learn new things in my job	○	○	○	○	○
I get to do a variety of different things in my job	○	○	○	○	○
I have an opportunity to develop my personal special abilities	○	○	○	○	○

41. Discrimination means treating people differently (without an acceptable reason) and placing them in an unfavourable position because they belong to a certain group. Have you personally experienced discrimination in your workplace in the past 12 months?

	Rarely or never	Not often	Sometimes	Fairly often	Very often or continuously
From a superior or management?	○	○	○	○	○

From co-workers?	○	○	○	○	○
From patients/clients?	○	○	○	○	○

JOB SATISFACTION AND WORK MOTIVATION

42. The following statements are about how you may see yourself as a nursing professional.

Nursing professionals who know me...

	Fully disagree	Somewhat disagree	Neither agree nor disagree	Somewhat agree	Fully agree
Respect my way of working	○	○	○	○	○
Respect my work-related ideas	○	○	○	○	○
Value my contribution to the work	○	○	○	○	○
Value me as a nursing professional	○	○	○	○	○
Think it would be difficult to replace me	○	○	○	○	○

43. Intention to change jobs

	No	Possibly	Yes
Have you planned changing jobs/employers?	○	○	○
Have you had plans for moving to work in another country in the next 12 months?	○	○	○

WORKING IN FINLAND

44. Have the following factors prevented you from getting a job you wanted or made getting it more complicated, or prevented your from working in Finland during the past two years?

	Not at all	Some	A lot
Lack of language skills (Finnish/Swedish)	○	○	○
Inadequate or unsuitable training (e.g. your previous profession or training is not acknowledged in Finland)	○	○	○
Lack of work experience	○	○	○
Employer attitudes	○	○	○
It is difficult to find information about vacancies	○	○	○
Insufficient relationships with people of Finnish origin who could help you find a job	○	○	○
Difficulty in finding housing	○	○	○
Your own uncertainty and fears related to starting a job	○	○	○
Adherence to a religion or belief	○	○	○
Reconciling work and family life	○	○	○
Other, please specify. ________________________________	○	○	○

45. Have the following factors promoted your employment or participation in working life in Finland during the last two years?

	I have not participated	I have participated, but I haven't	I have participated and found it	I have participated and found it

		found it useful	somewhat useful	very useful
Integration training	○	○	○	○
Employment services	○	○	○	○
Employer-provided language training	○	○	○	○
Other than employer-provided language training	○	○	○	○
Vocational education in the workplace	○	○	○	○
Vocational education elsewhere than in the workplace	○	○	○	○
Other, please specify. ______________________	○	○	○	○

46. How often on average do you have challenges in communicating in Finnish in the following work-related situations?

	Rarely or never	Not often	Sometimes	Fairly often	Very often or continuously	**I do not need Finnish in my work**
Face-to-face communication with patients	○	○	○	○	○	○
Face-to-face communication with co-workers	○	○	○	○	○	○
Workplace meetings	○	○	○	○	○	○
Keeping up with professional literature	○	○	○	○	○	○

Recording data in patient information systems	○	○	○	○	○	○
Electronic communication	○	○	○	○	○	○
Communication over the phone	○	○	○	○	○	○

47. In the following section, please respond to these statements concerning how well your supervisors and co-workers have explained to you how your workplace works and what the usual procedures are.

	Poorly or not at all	Rather poorly	Fairly well	Very well
They have explained what your principal duties are in your job	○	○	○	○
They have explained the rules regarding working hours and absences	○	○	○	○
They have explained who or what body can help you with various matters in the workplace	○	○	○	○
They have explained what things you can talk to your supervisor about	○	○	○	○
They have told you how to find information about decisions and changes concerning your workplace	○	○	○	○
They have told you whom to talk to if you feel bullied in the workplace	○	○	○	○
They have helped and supported you with coping better in Finnish (or Swedish) in the workplace	○	○	○	○

48. Compared to your Finnish co-workers, are your possibilities for doing the following things...

	Worse	Equal	Better
Participating in the development of the workplace community and working practices?	○	○	○
Improving your competence and professional skills (e.g. further education)?	○	○	○

49. To what extent has each of the things listed below disturbed, worried or stressed you in your job in the past six months? For each statement, select the alternative that best describes your opinion.

	Hardly ever	Not often	Sometimes	Fairly often	Very often or continuously
Constant rush and pressure due to uncompleted work	○	○	○	○	○
Not enough time to perform work properly	○	○	○	○	○
Changes in electronic information systems	○	○	○	○	○
Troublesome, poorly functioning hardware or software	○	○	○	○	○
Personnel turnover, short-term temps	○	○	○	○	○
Patients' expectations frequently differ from those of health care personnel	○	○	○	○	○
Difficult patients who complain, blame and criticise	○	○	○	○	○
Patients who are unwilling to co-operate and passive	○	○	○	○	○
Your own insufficient professional skills and knowledge	○	○	○	○	○
Responsibility for patients	○	○	○	○	○
Working in a foreign language	○	○	○	○	○

Finnish workplace customs and culture	○	○	○	○	○
Other people do not understand my culture	○	○	○	○	○

LIVING IN FINLAND

In this section, we will ask about your arrival and life in Finland.

50. Are you a Finnish citizen?

○ Yes

○ No

51. Are you living in Finland permanently?

○ Yes

○ No

52. In what year did you (first) move to Finland?

____

53. Overall, how satisfied or dissatisfied are you with your life in Finland?

	0	1	2	3	4	5	6	7	8	9	10	
very dissatisfied	○	○	○	○	○	○	○	○	○	○	○	very satisfied

54. On what grounds were you granted a residence permit in Finland?

○ As an ethnic returnee (e.g., Estonian or Russian)

○ As the spouse or child of a native of Finland

○ As the spouse or child of an immigrant residing permanently in Finland

○ As an employment-based immigrant

○ As an asylum seeker

○ As a refugee

○ Other

HEALTH AND WELLBEING

55. What is your health status compared to others of your age?

○ Good

○ Rather good

○ Average

○ Rather poor

○ Poor

56. Assume that your work ability at its best has a value of 10 and 0 would mean that you could not work at all. How many points would you give to your current work ability?

	0	1	2	3	4	5	6	7	8	9	10	
very bad	○	○	○	○	○	○	○	○	○	○	○	very good

57. Stress means a situation when a person feels tense, restless, nervous, or anxious, or is unable to sleep at night because his or her mind is troubled all the time Do you currently feel this kind of stress?

- ○ Not at all
- ○ Just a little
- ○ Some
- ○ Quite a lot
- ○ Very much

58. The following questions relate to your well-being during the past few weeks. Please select the most suitable option for each statement.

	Not at all	Not more than usual	Somewhat more than usual	Much more than usual
Have you recently lost much sleep over worry?	○	○	○	○
Have you recently felt constantly under strain?	○	○	○	○
Have you recently felt you couldn't overcome your difficulties?	○	○	○	○
Have you recently been feeling unhappy and depressed?	○	○	○	○

59. How often during the last four weeks have you had the following symptoms?

	Not at all	1-3 nights/month	Approximately one night/week	2-4 nights/week	5-6 nights/week	Every night
Difficulty falling asleep	○	○	○	○	○	○

Waking up several times per night	○	○	○	○	○	○
Having trouble staying asleep (including waking up too early)	○	○	○	○	○	○
Waking up after your usual amount of sleep feeling tired and worn out	○	○	○	○	○	○

60. In the space below you can type in your observations and opinions about this study and your experiences of working in Finland. Thank you for your responses!

____

____

____

[Break]

0% completed
