# Supplementary material for: The association between cross-cultural competence and well-being among registered native and foreign-born nurses in Finland
Source: PLoS One. 2018 Dec 7;13(12):e0208761. doi: 10.1371/journal.pone.0208761 (PMC6285347; doi:10.1371/journal.pone.0208761)
Supplement: S1 Table — (DOCX) [file pone.0208761.s004.docx]

S1 Table

*Fit of Measurement Invariance Models*

| Models | df | χ² | Δdf | Δχ² | Δχ² *p*-value | AIC | BIC | RMSEA | CFI | ΔRMSEA | ΔCFI |
| --- | --- | --- | --- | --- | --- | --- | --- | --- | --- | --- | --- |
| Configural | 396 | 949.12 |  |  |  | 43581 | 44309 | 0.058 | 0.918 |  |  |
| Metric | 414 | 973.48 | 18 | 24.36 | .144 | 43569 | 44213 | 0.057 | 0.917 | 0.001 | 0.001 |
| Scalar | 432 | 1087.72 | 18 | 114.23 | < .001 | 43648 | 44206 | 0.060 | 0.902 | 0.003 | 0.014 |
| Residual | 454 | 1194.60 | 22 | 106.89 | < .001 | 43711 | 44165 | 0.062 | 0.890 | 0.002 | 0.013 |
| Means | 458 | 1308.41 | 4 | 113.81 | < .001 | 43816 | 44251 | 0.067 | 0.873 | 0.004 | 0.016 |
